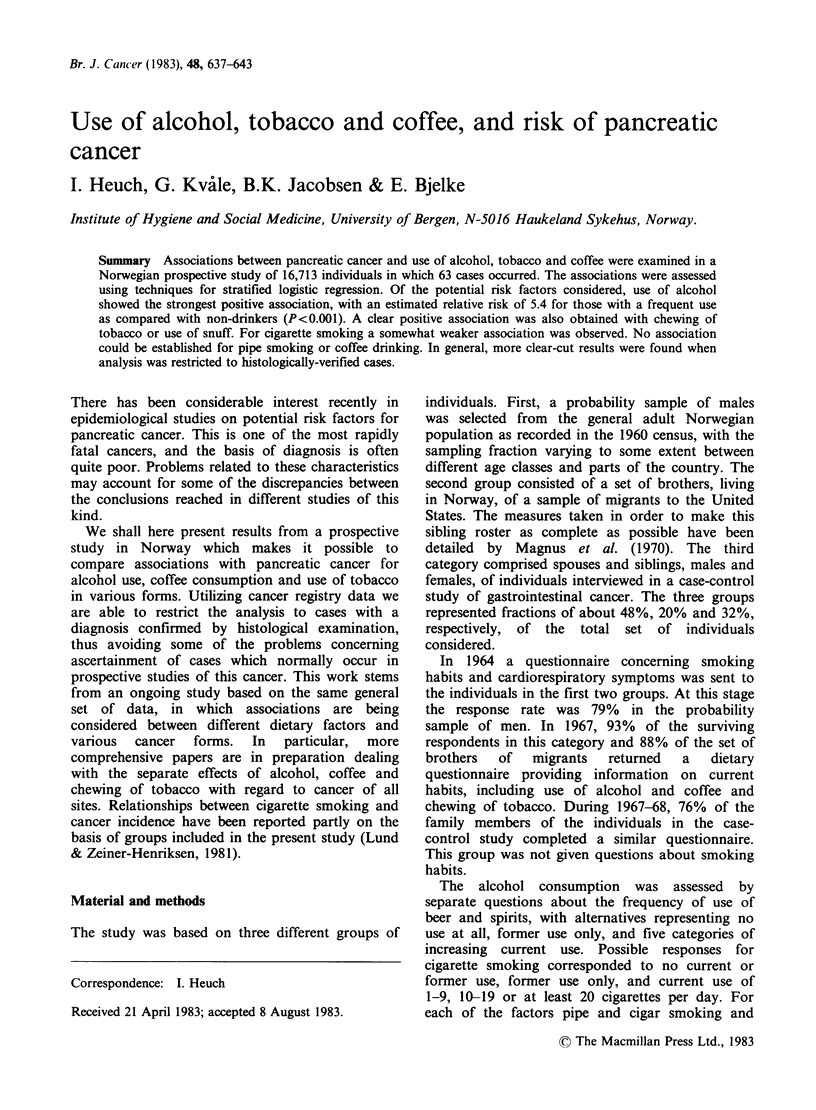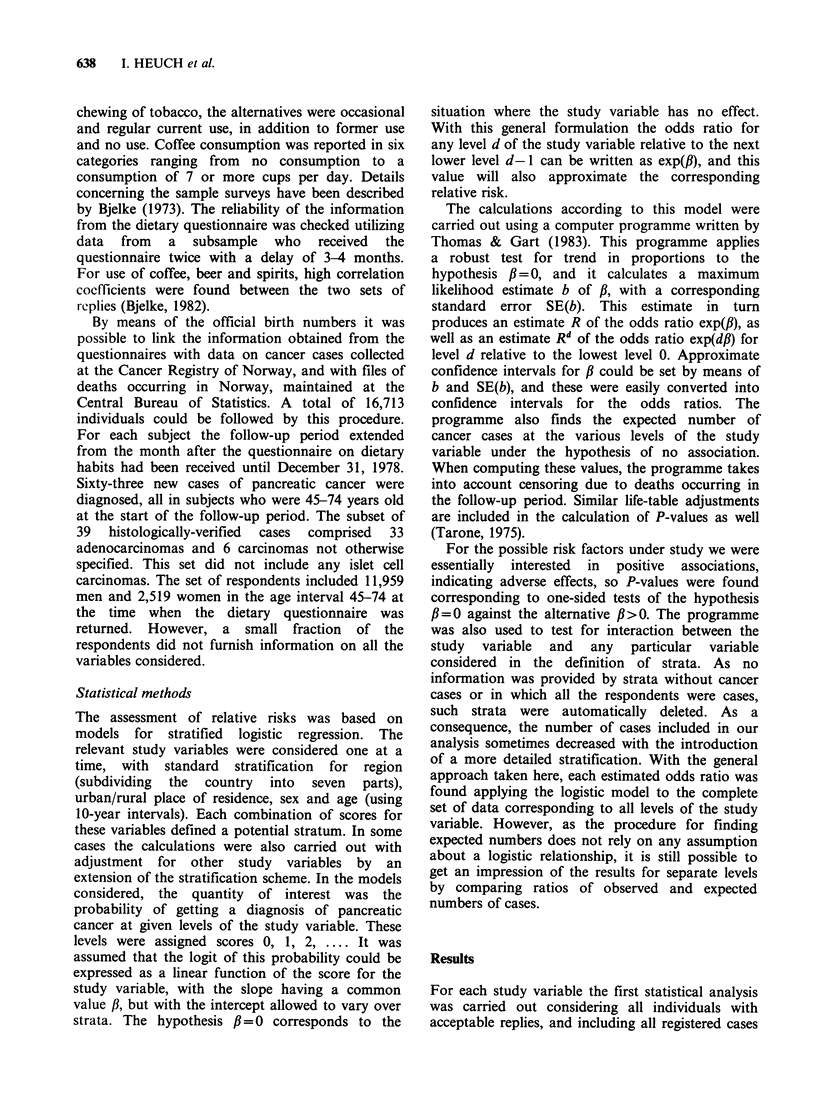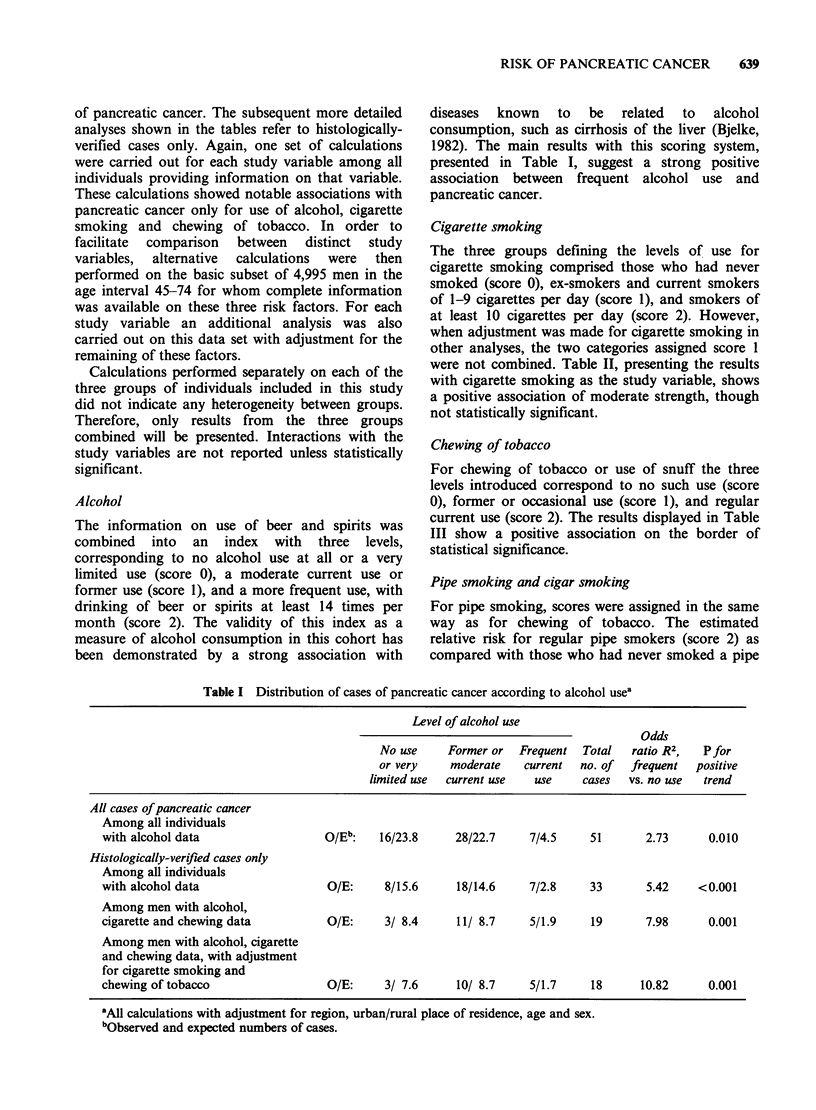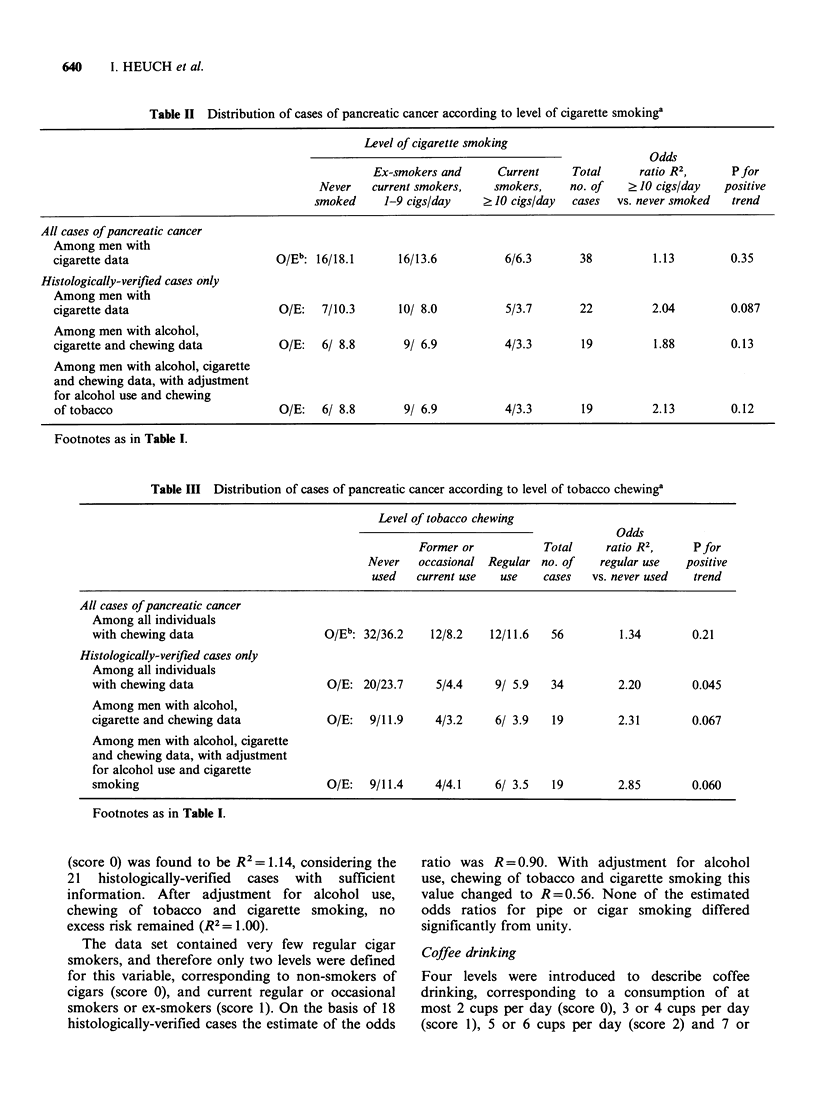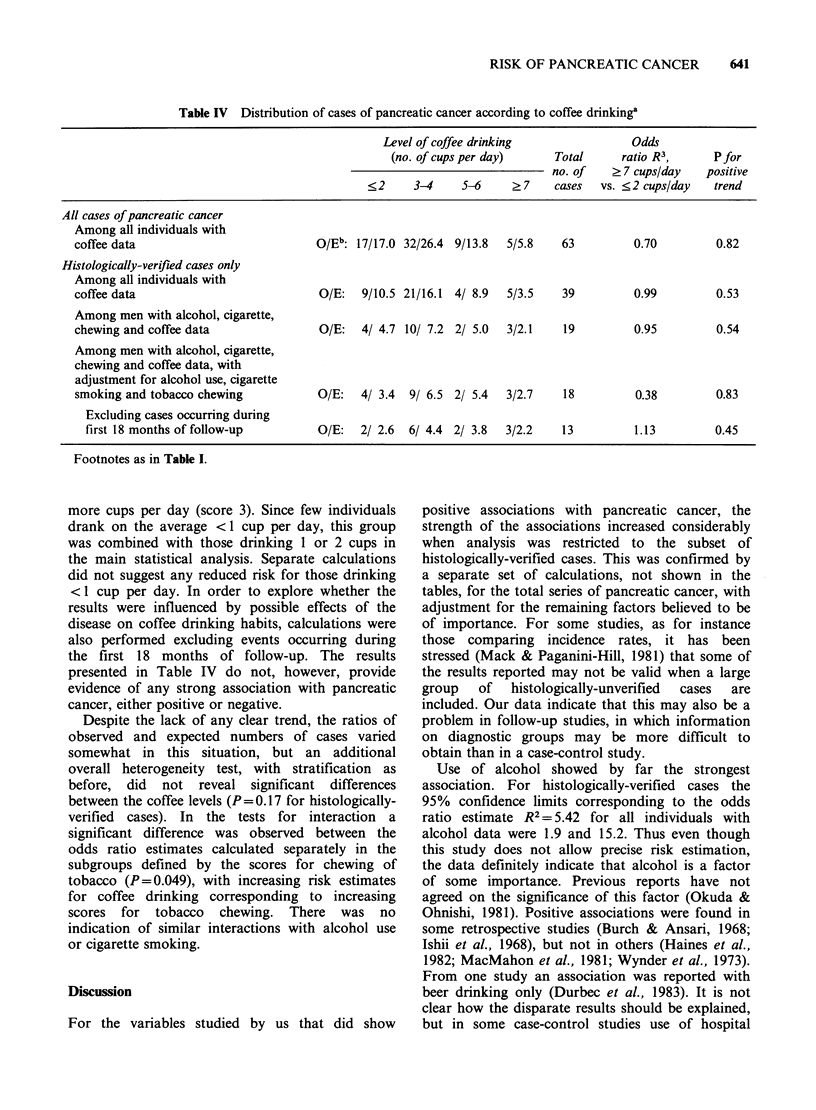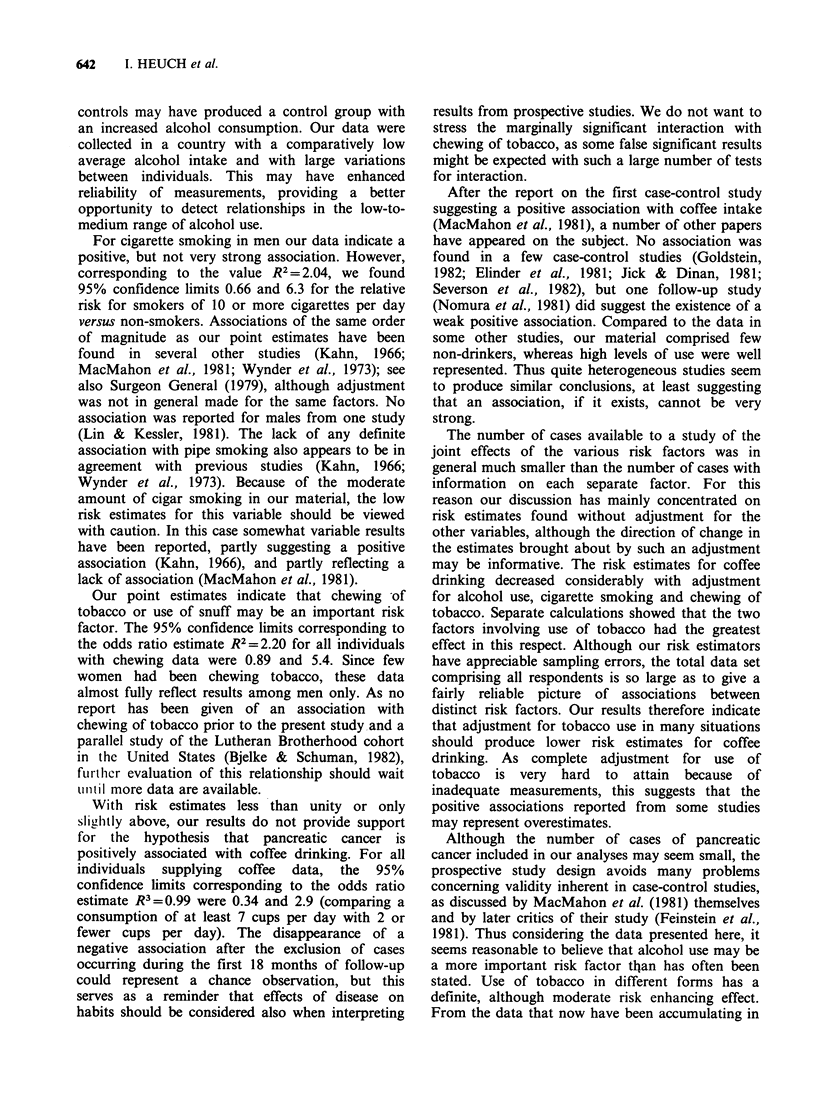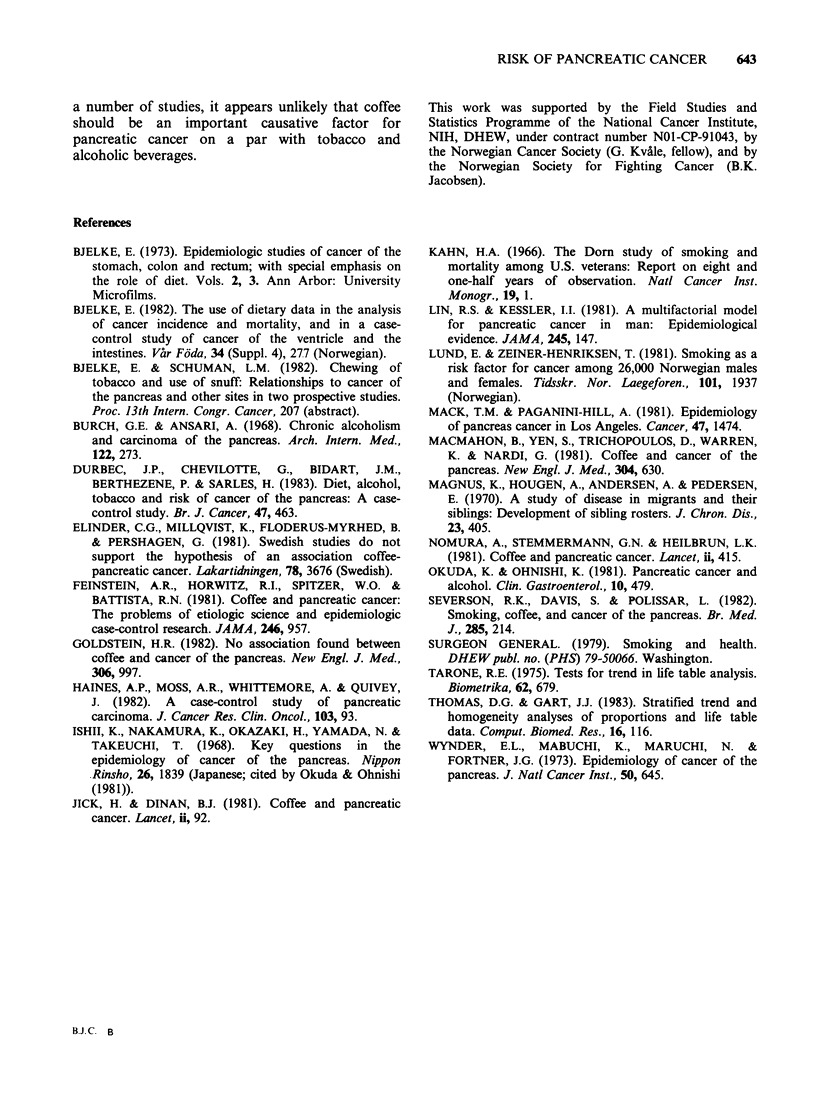# Use of alcohol, tobacco and coffee, and risk of pancreatic cancer.

**DOI:** 10.1038/bjc.1983.245

**Published:** 1983-11

**Authors:** I. Heuch, G. Kvåle, B. K. Jacobsen, E. Bjelke

## Abstract

Associations between pancreatic cancer and use of alcohol, tobacco and coffee were examined in a Norwegian prospective study of 16,713 individuals in which 63 cases occurred. The associations were assessed using techniques for stratified logistic regression. Of the potential risk factors considered, use of alcohol showed the strongest positive association, with an estimated relative risk of 5.4 for those with a frequent use as compared with non-drinkers (P less than 0.001). A clear positive association was also obtained with chewing of tobacco or use of snuff. For cigarette smoking a somewhat weaker association was observed. No association could be established for pipe smoking or coffee drinking. In general, more clear-cut results were found when analysis was restricted to histologically-verified cases.


Br. J. Cancer (1983), **48**, 637–643

### Use of alcohol, tobacco and coffee, and risk of pancreatic cancer

I. Heuch, G. Kvåle, B.K. Jacobsen & E. Bjelke

*Institute of Hygiene and Social Medicine, University of Bergen, N-5016 Haukeland Sykehus, Norway.*

**Summary** Associations between pancreatic cancer and use of alcohol, tobacco and coffee were examined in a Norwegian prospective study of 16,713 individuals in which 63 cases occurred. The associations were assessed using techniques for stratified logistic regression. Of the potential risk factors considered, use of alcohol showed the strongest positive association, with an estimated relative risk of 5.4 for those with a frequent use as compared with non-drinkers ($P<0.001$). A clear positive association was also obtained with chewing of tobacco or use of snuff. For cigarette smoking a somewhat weaker association was observed. No association could be established for pipe smoking or coffee drinking. In general, more clear-cut results were found when analysis was restricted to histologically-verified cases.

There has been considerable interest recently in epidemiological studies on potential risk factors for pancreatic cancer. This is one of the most rapidly fatal cancers, and the basis of diagnosis is often quite poor. Problems related to these characteristics may account for some of the discrepancies between the conclusions reached in different studies of this kind.

We shall here present results from a prospective study in Norway which makes it possible to compare associations with pancreatic cancer for alcohol use, coffee consumption and use of tobacco in various forms. Utilizing cancer registry data we are able to restrict the analysis to cases with a diagnosis confirmed by histological examination, thus avoiding some of the problems concerning ascertainment of cases which normally occur in prospective studies of this cancer. This work stems from an ongoing study based on the same general set of data, in which associations are being considered between different dietary factors and various cancer forms. In particular, more comprehensive papers are in preparation dealing with the separate effects of alcohol, coffee and chewing of tobacco with regard to cancer of all sites. Relationships between cigarette smoking and cancer incidence have been reported partly on the basis of groups included in the present study (Lund & Zeiner-Henriksen, 1981).

### Material and methods

The study was based on three different groups of individuals. First, a probability sample of males was selected from the general adult Norwegian population as recorded in the 1960 census, with the sampling fraction varying to some extent between different age classes and parts of the country. The second group consisted of a set of brothers, living in Norway, of a sample of migrants to the United States. The measures taken in order to make this sibling roster as complete as possible have been detailed by Magnus *et al.* (1970). The third category comprised spouses and siblings, males and females, of individuals interviewed in a case-control study of gastrointestinal cancer. The three groups represented fractions of about 48%, 20% and 32%, respectively, of the total set of individuals considered.

In 1964 a questionnaire concerning smoking habits and cardiorespiratory symptoms was sent to the individuals in the first two groups. At this stage the response rate was 79% in the probability sample of men. In 1967, 93% of the surviving respondents in this category and 88% of the set of brothers of migrants returned a dietary questionnaire providing information on current habits, including use of alcohol and coffee and chewing of tobacco. During 1967–68, 76% of the family members of the individuals in the case-control study completed a similar questionnaire. This group was not given questions about smoking habits.

The alcohol consumption was assessed by separate questions about the frequency of use of beer and spirits, with alternatives representing no use at all, former use only, and five categories of increasing current use. Possible responses for cigarette smoking corresponded to no current or former use, former use only, and current use of 1–9, 10–19 or at least 20 cigarettes per day. For each of the factors pipe and cigar smoking and

Correspondence: I. Heuch

Received 21 April 1983; accepted 8 August 1983.

© The Macmillan Press Ltd., 1983

chewing of tobacco, the alternatives were occasional and regular current use, in addition to former use and no use. Coffee consumption was reported in six categories ranging from no consumption to a consumption of 7 or more cups per day. Details concerning the sample surveys have been described by Bjelke (1973). The reliability of the information from the dietary questionnaire was checked utilizing data from a subsample who received the questionnaire twice with a delay of 3–4 months. For use of coffee, beer and spirits, high correlation coefficients were found between the two sets of replies (Bjelke, 1982).

By means of the official birth numbers it was possible to link the information obtained from the questionnaires with data on cancer cases collected at the Cancer Registry of Norway, and with files of deaths occurring in Norway, maintained at the Central Bureau of Statistics. A total of 16,713 individuals could be followed by this procedure. For each subject the follow-up period extended from the month after the questionnaire on dietary habits had been received until December 31, 1978. Sixty-three new cases of pancreatic cancer were diagnosed, all in subjects who were 45–74 years old at the start of the follow-up period. The subset of 39 histologically-verified cases comprised 33 adenocarcinomas and 6 carcinomas not otherwise specified. This set did not include any islet cell carcinomas. The set of respondents included 11,959 men and 2,519 women in the age interval 45–74 at the time when the dietary questionnaire was returned. However, a small fraction of the respondents did not furnish information on all the variables considered.

### *Statistical methods*

The assessment of relative risks was based on models for stratified logistic regression. The relevant study variables were considered one at a time, with standard stratification for region (subdividing the country into seven parts), urban/rural place of residence, sex and age (using 10-year intervals). Each combination of scores for these variables defined a potential stratum. In some cases the calculations were also carried out with adjustment for other study variables by an extension of the stratification scheme. In the models considered, the quantity of interest was the probability of getting a diagnosis of pancreatic cancer at given levels of the study variable. These levels were assigned scores 0, 1, 2, .... It was assumed that the logit of this probability could be expressed as a linear function of the score for the study variable, with the slope having a common value $\beta$, but with the intercept allowed to vary over strata. The hypothesis $\beta=0$ corresponds to the situation where the study variable has no effect. With this general formulation the odds ratio for any level $d$ of the study variable relative to the next lower level $d-1$ can be written as $\exp(\beta)$, and this value will also approximate the corresponding relative risk.

The calculations according to this model were carried out using a computer programme written by Thomas & Gart (1983). This programme applies a robust test for trend in proportions to the hypothesis $\beta=0$, and it calculates a maximum likelihood estimate $b$ of $\beta$, with a corresponding standard error SE($b$). This estimate in turn produces an estimate $R$ of the odds ratio $\exp(\beta)$, as well as an estimate $R^d$ of the odds ratio $\exp(d\beta)$ for level $d$ relative to the lowest level 0. Approximate confidence intervals for $\beta$ could be set by means of $b$ and SE($b$), and these were easily converted into confidence intervals for the odds ratios. The programme also finds the expected number of cancer cases at the various levels of the study variable under the hypothesis of no association. When computing these values, the programme takes into account censoring due to deaths occurring in the follow-up period. Similar life-table adjustments are included in the calculation of $P$-values as well (Tarone, 1975).

For the possible risk factors under study we were essentially interested in positive associations, indicating adverse effects, so $P$-values were found corresponding to one-sided tests of the hypothesis $\beta=0$ against the alternative $\beta>0$. The programme was also used to test for interaction between the study variable and any particular variable considered in the definition of strata. As no information was provided by strata without cancer cases or in which all the respondents were cases, such strata were automatically deleted. As a consequence, the number of cases included in our analysis sometimes decreased with the introduction of a more detailed stratification. With the general approach taken here, each estimated odds ratio was found applying the logistic model to the complete set of data corresponding to all levels of the study variable. However, as the procedure for finding expected numbers does not rely on any assumption about a logistic relationship, it is still possible to get an impression of the results for separate levels by comparing ratios of observed and expected numbers of cases.

### Results

For each study variable the first statistical analysis was carried out considering all individuals with acceptable replies, and including all registered cases of pancreatic cancer. The subsequent more detailed analyses shown in the tables refer to histologically-verified cases only. Again, one set of calculations were carried out for each study variable among all individuals providing information on that variable. These calculations showed notable associations with pancreatic cancer only for use of alcohol, cigarette smoking and chewing of tobacco. In order to facilitate comparison between distinct study variables, alternative calculations were then performed on the basic subset of 4,995 men in the age interval 45–74 for whom complete information was available on these three risk factors. For each study variable an additional analysis was also carried out on this data set with adjustment for the remaining of these factors.

Calculations performed separately on each of the three groups of individuals included in this study did not indicate any heterogeneity between groups. Therefore, only results from the three groups combined will be presented. Interactions with the study variables are not reported unless statistically significant.

*Alcohol*

The information on use of beer and spirits was combined into an index with three levels, corresponding to no alcohol use at all or a very limited use (score 0), a moderate current use or former use (score 1), and a more frequent use, with drinking of beer or spirits at least 14 times per month (score 2). The validity of this index as a measure of alcohol consumption in this cohort has been demonstrated by a strong association with diseases known to be related to alcohol consumption, such as cirrhosis of the liver (Bjelke, 1982). The main results with this scoring system, presented in Table I, suggest a strong positive association between frequent alcohol use and pancreatic cancer.

*Cigarette smoking*

The three groups defining the levels of use for cigarette smoking comprised those who had never smoked (score 0), ex-smokers and current smokers of 1–9 cigarettes per day (score 1), and smokers of at least 10 cigarettes per day (score 2). However, when adjustment was made for cigarette smoking in other analyses, the two categories assigned score 1 were not combined. Table II, presenting the results with cigarette smoking as the study variable, shows a positive association of moderate strength, though not statistically significant.

*Chewing of tobacco*

For chewing of tobacco or use of snuff the three levels introduced correspond to no such use (score 0), former or occasional use (score 1), and regular current use (score 2). The results displayed in Table III show a positive association on the border of statistical significance.

*Pipe smoking and cigar smoking*

For pipe smoking, scores were assigned in the same way as for chewing of tobacco. The estimated relative risk for regular pipe smokers (score 2) as compared with those who had never smoked a pipe

**Table I** Distribution of cases of pancreatic cancer according to alcohol use[a]

| | | *Level of alcohol use* | | | | | |
|---|---|---|---|---|---|---|---|
| | | *No use or very limited use* | *Former or moderate current use* | *Frequent current use* | *Total no. of cases* | *Odds ratio $R^2$, frequent vs. no use* | P *for positive trend* |
| *All cases of pancreatic cancer* | | | | | | | |
| Among all individuals with alcohol data | O/E[b]: | 16/23.8 | 28/22.7 | 7/4.5 | 51 | 2.73 | 0.010 |
| *Histologically-verified cases only* | | | | | | | |
| Among all individuals with alcohol data | O/E: | 8/15.6 | 18/14.6 | 7/2.8 | 33 | 5.42 | <0.001 |
| Among men with alcohol, cigarette and chewing data | O/E: | 3/ 8.4 | 11/ 8.7 | 5/1.9 | 19 | 7.98 | 0.001 |
| Among men with alcohol, cigarette and chewing data, with adjustment for cigarette smoking and chewing of tobacco | O/E: | 3/ 7.6 | 10/ 8.7 | 5/1.7 | 18 | 10.82 | 0.001 |

[a]All calculations with adjustment for region, urban/rural place of residence, age and sex.
[b]Observed and expected numbers of cases.

**Table II** Distribution of cases of pancreatic cancer according to level of cigarette smoking[a]

| | | Level of cigarette smoking | | | | | |
|---|---|---|---|---|---|---|---|
| | | *Never smoked* | *Ex-smokers and current smokers, 1–9 cigs/day* | *Current smokers, ≥10 cigs/day* | *Total no. of cases* | *Odds ratio $R^2$, ≥10 cigs/day vs. never smoked* | P *for positive trend* |
| *All cases of pancreatic cancer* | | | | | | | |
| Among men with cigarette data | O/E[b]: | 16/18.1 | 16/13.6 | 6/6.3 | 38 | 1.13 | 0.35 |
| *Histologically-verified cases only* | | | | | | | |
| Among men with cigarette data | O/E: | 7/10.3 | 10/ 8.0 | 5/3.7 | 22 | 2.04 | 0.087 |
| Among men with alcohol, cigarette and chewing data | O/E: | 6/ 8.8 | 9/ 6.9 | 4/3.3 | 19 | 1.88 | 0.13 |
| Among men with alcohol, cigarette and chewing data, with adjustment for alcohol use and chewing of tobacco | O/E: | 6/ 8.8 | 9/ 6.9 | 4/3.3 | 19 | 2.13 | 0.12 |

Footnotes as in **Table I**.

**Table III** Distribution of cases of pancreatic cancer according to level of tobacco chewing[a]

| | | Level of tobacco chewing | | | | | |
|---|---|---|---|---|---|---|---|
| | | *Never used* | *Former or occasional current use* | *Regular use* | *Total no. of cases* | *Odds ratio $R^2$, regular use vs. never used* | P *for positive trend* |
| *All cases of pancreatic cancer* | | | | | | | |
| Among all individuals with chewing data | O/E[b]: | 32/36.2 | 12/8.2 | 12/11.6 | 56 | 1.34 | 0.21 |
| *Histologically-verified cases only* | | | | | | | |
| Among all individuals with chewing data | O/E: | 20/23.7 | 5/4.4 | 9/ 5.9 | 34 | 2.20 | 0.045 |
| Among men with alcohol, cigarette and chewing data | O/E: | 9/11.9 | 4/3.2 | 6/ 3.9 | 19 | 2.31 | 0.067 |
| Among men with alcohol, cigarette and chewing data, with adjustment for alcohol use and cigarette smoking | O/E: | 9/11.4 | 4/4.1 | 6/ 3.5 | 19 | 2.85 | 0.060 |

Footnotes as in **Table I**.

(score 0) was found to be $R^2 = 1.14$, considering the 21 histologically-verified cases with sufficient information. After adjustment for alcohol use, chewing of tobacco and cigarette smoking, no excess risk remained ($R^2 = 1.00$).

The data set contained very few regular cigar smokers, and therefore only two levels were defined for this variable, corresponding to non-smokers of cigars (score 0), and current regular or occasional smokers or ex-smokers (score 1). On the basis of 18 histologically-verified cases the estimate of the odds ratio was $R = 0.90$. With adjustment for alcohol use, chewing of tobacco and cigarette smoking this value changed to $R = 0.56$. None of the estimated odds ratios for pipe or cigar smoking differed significantly from unity.

*Coffee drinking*

Four levels were introduced to describe coffee drinking, corresponding to a consumption of at most 2 cups per day (score 0), 3 or 4 cups per day (score 1), 5 or 6 cups per day (score 2) and 7 or

**Table IV** Distribution of cases of pancreatic cancer according to coffee drinking[a]

| | | Level of coffee drinking (no. of cups per day) | | | | Total no. of cases | Odds ratio $R^3$, ≥7 cups/day vs. ≤2 cups/day | P for positive trend |
|---|---|---|---|---|---|---|---|---|
| | | ≤2 | 3–4 | 5–6 | ≥7 | | | |
| *All cases of pancreatic cancer* | | | | | | | | |
| Among all individuals with coffee data | O/E[b]: | 17/17.0 | 32/26.4 | 9/13.8 | 5/5.8 | 63 | 0.70 | 0.82 |
| *Histologically-verified cases only* | | | | | | | | |
| Among all individuals with coffee data | O/E: | 9/10.5 | 21/16.1 | 4/ 8.9 | 5/3.5 | 39 | 0.99 | 0.53 |
| Among men with alcohol, cigarette, chewing and coffee data | O/E: | 4/ 4.7 | 10/ 7.2 | 2/ 5.0 | 3/2.1 | 19 | 0.95 | 0.54 |
| Among men with alcohol, cigarette, chewing and coffee data, with adjustment for alcohol use, cigarette smoking and tobacco chewing | O/E: | 4/ 3.4 | 9/ 6.5 | 2/ 5.4 | 3/2.7 | 18 | 0.38 | 0.83 |
| Excluding cases occurring during first 18 months of follow-up | O/E: | 2/ 2.6 | 6/ 4.4 | 2/ 3.8 | 3/2.2 | 13 | 1.13 | 0.45 |

Footnotes as in **Table I**.

more cups per day (score 3). Since few individuals drank on the average <1 cup per day, this group was combined with those drinking 1 or 2 cups in the main statistical analysis. Separate calculations did not suggest any reduced risk for those drinking <1 cup per day. In order to explore whether the results were influenced by possible effects of the disease on coffee drinking habits, calculations were also performed excluding events occurring during the first 18 months of follow-up. The results presented in Table IV do not, however, provide evidence of any strong association with pancreatic cancer, either positive or negative.

Despite the lack of any clear trend, the ratios of observed and expected numbers of cases varied somewhat in this situation, but an additional overall heterogeneity test, with stratification as before, did not reveal significant differences between the coffee levels ($P=0.17$ for histologically-verified cases). In the tests for interaction a significant difference was observed between the odds ratio estimates calculated separately in the subgroups defined by the scores for chewing of tobacco ($P=0.049$), with increasing risk estimates for coffee drinking corresponding to increasing scores for tobacco chewing. There was no indication of similar interactions with alcohol use or cigarette smoking.

### Discussion

For the variables studied by us that did show positive associations with pancreatic cancer, the strength of the associations increased considerably when analysis was restricted to the subset of histologically-verified cases. This was confirmed by a separate set of calculations, not shown in the tables, for the total series of pancreatic cancer, with adjustment for the remaining factors believed to be of importance. For some studies, as for instance those comparing incidence rates, it has been stressed (Mack & Paganini-Hill, 1981) that some of the results reported may not be valid when a large group of histologically-unverified cases are included. Our data indicate that this may also be a problem in follow-up studies, in which information on diagnostic groups may be more difficult to obtain than in a case-control study.

Use of alcohol showed by far the strongest association. For histologically-verified cases the 95% confidence limits corresponding to the odds ratio estimate $R^2=5.42$ for all individuals with alcohol data were 1.9 and 15.2. Thus even though this study does not allow precise risk estimation, the data definitely indicate that alcohol is a factor of some importance. Previous reports have not agreed on the significance of this factor (Okuda & Ohnishi, 1981). Positive associations were found in some retrospective studies (Burch & Ansari, 1968; Ishii *et al.*, 1968), but not in others (Haines *et al.*, 1982; MacMahon *et al.*, 1981; Wynder *et al.*, 1973). From one study an association was reported with beer drinking only (Durbec *et al.*, 1983). It is not clear how the disparate results should be explained, but in some case-control studies use of hospital controls may have produced a control group with an increased alcohol consumption. Our data were collected in a country with a comparatively low average alcohol intake and with large variations between individuals. This may have enhanced reliability of measurements, providing a better opportunity to detect relationships in the low-to-medium range of alcohol use.

For cigarette smoking in men our data indicate a positive, but not very strong association. However, corresponding to the value $R^2 = 2.04$, we found 95% confidence limits 0.66 and 6.3 for the relative risk for smokers of 10 or more cigarettes per day *versus* non-smokers. Associations of the same order of magnitude as our point estimates have been found in several other studies (Kahn, 1966; MacMahon *et al.*, 1981; Wynder *et al.*, 1973); see also Surgeon General (1979), although adjustment was not in general made for the same factors. No association was reported for males from one study (Lin & Kessler, 1981). The lack of any definite association with pipe smoking also appears to be in agreement with previous studies (Kahn, 1966; Wynder *et al.*, 1973). Because of the moderate amount of cigar smoking in our material, the low risk estimates for this variable should be viewed with caution. In this case somewhat variable results have been reported, partly suggesting a positive association (Kahn, 1966), and partly reflecting a lack of association (MacMahon *et al.*, 1981).

Our point estimates indicate that chewing of tobacco or use of snuff may be an important risk factor. The 95% confidence limits corresponding to the odds ratio estimate $R^2 = 2.20$ for all individuals with chewing data were 0.89 and 5.4. Since few women had been chewing tobacco, these data almost fully reflect results among men only. As no report has been given of an association with chewing of tobacco prior to the present study and a parallel study of the Lutheran Brotherhood cohort in the United States (Bjelke & Schuman, 1982), further evaluation of this relationship should wait until more data are available.

With risk estimates less than unity or only slightly above, our results do not provide support for the hypothesis that pancreatic cancer is positively associated with coffee drinking. For all individuals supplying coffee data, the 95% confidence limits corresponding to the odds ratio estimate $R^3 = 0.99$ were 0.34 and 2.9 (comparing a consumption of at least 7 cups per day with 2 or fewer cups per day). The disappearance of a negative association after the exclusion of cases occurring during the first 18 months of follow-up could represent a chance observation, but this serves as a reminder that effects of disease on habits should be considered also when interpreting results from prospective studies. We do not want to stress the marginally significant interaction with chewing of tobacco, as some false significant results might be expected with such a large number of tests for interaction.

After the report on the first case-control study suggesting a positive association with coffee intake (MacMahon *et al.*, 1981), a number of other papers have appeared on the subject. No association was found in a few case-control studies (Goldstein, 1982; Elinder *et al.*, 1981; Jick & Dinan, 1981; Severson *et al.*, 1982), but one follow-up study (Nomura *et al.*, 1981) did suggest the existence of a weak positive association. Compared to the data in some other studies, our material comprised few non-drinkers, whereas high levels of use were well represented. Thus quite heterogeneous studies seem to produce similar conclusions, at least suggesting that an association, if it exists, cannot be very strong.

The number of cases available to a study of the joint effects of the various risk factors was in general much smaller than the number of cases with information on each separate factor. For this reason our discussion has mainly concentrated on risk estimates found without adjustment for the other variables, although the direction of change in the estimates brought about by such an adjustment may be informative. The risk estimates for coffee drinking decreased considerably with adjustment for alcohol use, cigarette smoking and chewing of tobacco. Separate calculations showed that the two factors involving use of tobacco had the greatest effect in this respect. Although our risk estimators have appreciable sampling errors, the total data set comprising all respondents is so large as to give a fairly reliable picture of associations between distinct risk factors. Our results therefore indicate that adjustment for tobacco use in many situations should produce lower risk estimates for coffee drinking. As complete adjustment for use of tobacco is very hard to attain because of inadequate measurements, this suggests that the positive associations reported from some studies may represent overestimates.

Although the number of cases of pancreatic cancer included in our analyses may seem small, the prospective study design avoids many problems concerning validity inherent in case-control studies, as discussed by MacMahon *et al.* (1981) themselves and by later critics of their study (Feinstein *et al.*, 1981). Thus considering the data presented here, it seems reasonable to believe that alcohol use may be a more important risk factor than has often been stated. Use of tobacco in different forms has a definite, although moderate risk enhancing effect. From the data that now have been accumulating in a number of studies, it appears unlikely that coffee should be an important causative factor for pancreatic cancer on a par with tobacco and alcoholic beverages.

This work was supported by the Field Studies and Statistics Programme of the National Cancer Institute, NIH, DHEW, under contract number N01-CP-91043, by the Norwegian Cancer Society (G. Kvåle, fellow), and by the Norwegian Society for Fighting Cancer (B.K. Jacobsen).

### References

BJELKE, E. (1973). Epidemiologic studies of cancer of the stomach, colon and rectum; with special emphasis on the role of diet. Vols. **2**, **3**. Ann Arbor: University Microfilms.

BJELKE, E. (1982). The use of dietary data in the analysis of cancer incidence and mortality, and in a case-control study of cancer of the ventricle and the intestines. *Vår Föda*, **34** (Suppl. 4), 277 (Norwegian).

BJELKE, E. & SCHUMAN, L.M. (1982). Chewing of tobacco and use of snuff: Relationships to cancer of the pancreas and other sites in two prospective studies. *Proc. 13th Intern. Congr. Cancer*, 207 (abstract).

BURCH, G.E. & ANSARI, A. (1968). Chronic alcoholism and carcinoma of the pancreas. *Arch. Intern. Med.*, **122**, 273.

DURBEC, J.P., CHEVILOTTE, G., BIDART, J.M., BERTHEZENE, P. & SARLES, H. (1983). Diet, alcohol, tobacco and risk of cancer of the pancreas: A case-control study. *Br. J. Cancer*, **47**, 463.

ELINDER, C.G., MILLQVIST, K., FLODERUS-MYRHED, B. & PERSHAGEN, G. (1981). Swedish studies do not support the hypothesis of an association coffee-pancreatic cancer. *Lakartidningen*, **78**, 3676 (Swedish).

FEINSTEIN, A.R., HORWITZ, R.I., SPITZER, W.O. & BATTISTA, R.N. (1981). Coffee and pancreatic cancer: The problems of etiologic science and epidemiologic case-control research. *JAMA*, **246**, 957.

GOLDSTEIN, H.R. (1982). No association found between coffee and cancer of the pancreas. *New Engl. J. Med.*, **306**, 997.

HAINES, A.P., MOSS, A.R., WHITTEMORE, A. & QUIVEY, J. (1982). A case-control study of pancreatic carcinoma. *J. Cancer Res. Clin. Oncol.*, **103**, 93.

ISHII, K., NAKAMURA, K., OKAZAKI, H., YAMADA, N. & TAKEUCHI, T. (1968). Key questions in the epidemiology of cancer of the pancreas. *Nippon Rinsho*, **26**, 1839 (Japanese; cited by Okuda & Ohnishi (1981)).

JICK, H. & DINAN, B.J. (1981). Coffee and pancreatic cancer. *Lancet*, **ii**, 92.

KAHN, H.A. (1966). The Dorn study of smoking and mortality among U.S. veterans: Report on eight and one-half years of observation. *Natl Cancer Inst. Monogr.*, **19**, 1.

LIN, R.S. & KESSLER, I.I. (1981). A multifactorial model for pancreatic cancer in man: Epidemiological evidence. *JAMA*, **245**, 147.

LUND, E. & ZEINER-HENRIKSEN, T. (1981). Smoking as a risk factor for cancer among 26,000 Norwegian males and females. *Tidsskr. Nor. Laegeforen.*, **101**, 1937 (Norwegian).

MACK, T.M. & PAGANINI-HILL, A. (1981). Epidemiology of pancreas cancer in Los Angeles. *Cancer*, **47**, 1474.

MACMAHON, B., YEN, S., TRICHOPOULOS, D., WARREN, K. & NARDI, G. (1981). Coffee and cancer of the pancreas. *New Engl. J. Med.*, **304**, 630.

MAGNUS, K., HOUGEN, A., ANDERSEN, A. & PEDERSEN, E. (1970). A study of disease in migrants and their siblings: Development of sibling rosters. *J. Chron. Dis.*, **23**, 405.

NOMURA, A., STEMMERMANN, G.N. & HEILBRUN, L.K. (1981). Coffee and pancreatic cancer. *Lancet*, **ii**, 415.

OKUDA, K. & OHNISHI, K. (1981). Pancreatic cancer and alcohol. *Clin. Gastroenterol.*, **10**, 479.

SEVERSON, R.K., DAVIS, S. & POLISSAR, L. (1982). Smoking, coffee, and cancer of the pancreas. *Br. Med. J.*, **285**, 214.

SURGEON GENERAL. (1979). Smoking and health. *DHEW publ. no. (PHS) 79-50066*. Washington.

TARONE, R.E. (1975). Tests for trend in life table analysis. *Biometrika*, **62**, 679.

THOMAS, D.G. & GART, J.J. (1983). Stratified trend and homogeneity analyses of proportions and life table data. *Comput. Biomed. Res.*, **16**, 116.

WYNDER, E.L., MABUCHI, K., MARUCHI, N. & FORTNER, J.G. (1973). Epidemiology of cancer of the pancreas. *J. Natl Cancer Inst.*, **50**, 645.